# Variations in older people’s emergency care use by social care setting: a systematic review of international evidence

**DOI:** 10.1093/bmb/ldad033

**Published:** 2023-12-18

**Authors:** Kelly Brotherhood, Ben Searle, Gemma Frances Spiers, Camila Caiado, Barbara Hanratty

**Affiliations:** Population Health Sciences Institute, Faculty of Medical Sciences, Newcastle University, Biomedical Research Building (Second Floor), Newcastle upon Tyne NE1 7RU, UK; Population Health Sciences Institute, Faculty of Medical Sciences, Newcastle University, Biomedical Research Building (Second Floor), Newcastle upon Tyne NE1 7RU, UK; Population Health Sciences Institute, Faculty of Medical Sciences, Newcastle University, Biomedical Research Building (Second Floor), Newcastle upon Tyne NE1 7RU, UK; Department of Mathematical Sciences, Mathematical Sciences & Computer Science Building, Durham University, Upper Mountjoy Campus, Stockton Road, Durham, DH1 3LE, UK; Population Health Sciences Institute, Faculty of Medical Sciences, Newcastle University, Biomedical Research Building (Second Floor), Newcastle upon Tyne NE1 7RU, UK

**Keywords:** older people, social care, emergency care, hospital attendance, hospital admissions

## Abstract

**Background:**

Older adults’ use of social care and their healthcare utilization are closely related. Residents of care homes access emergency care more often than the wider older population; however, less is known about emergency care use across other social care settings.

**Sources of data:**

A systematic review was conducted, searching six electronic databases between January 2012 and February 2022.

**Areas of agreement:**

Older people access emergency care from a variety of community settings.

**Areas of controversy:**

Differences in study design contributed to high variation observed between studies.

**Growing points:**

Although data were limited, findings suggest that emergency hospital attendance is lowest from nursing homes and highest from assisted living facilities, whilst emergency admissions varied little by social care setting.

**Areas timely for developing research:**

There is a paucity of published research on emergency hospital use from social care settings, particularly home care and assisted living facilities. More attention is needed on this area, with standardized definitions to enable comparisons between studies.

## Introduction

Demand for emergency care in England has risen over the past decade.^1^ The older population, who are living with an increasing number of complex care needs, has seen a larger increase in emergency hospital attendance in recent years than any other age group.^2–4^ Older people are also more likely to be admitted to hospital as an emergency after visiting the Emergency Department in comparison with other age groups.^5^ Whilst emergency hospital-based care is often the most appropriate method of administering care, visits to the Emergency Department can have adverse consequences for older people. Older people admitted to hospital are vulnerable to infection, face an increased risk of delirium and often see a decline in their health and wellbeing post-discharge from hospital.^6^ Therefore, it is important that older people attend the Emergency Department only when necessary.

Older people access emergency care from a variety of settings in the community. Care home residents’ use of emergency care is disproportionately high, in comparison with the wider older population.^7^ However, patterns of Emergency Department use are less clear from other social care settings. As the size of our older population grows,^8^ demand for social care is expected to rise, in terms of the number of people requiring care and the complexity of their care needs.^9^ This is particularly concerning in some regions of England, such as the North East, where the number of care home beds is declining.^10^ Here, home-based care and assisted living services will play a key role in supporting the needs of the older population.

Many visits to the Emergency Department made by the older population are thought to be for conditions that could potentially be managed outside of a hospital setting. To support policy efforts to reduce avoidable demand for emergency care, both in England and for many other Western governments, interventions must be timely and targeted at the right populations. Current approaches focus on delivering urgent healthcare in community settings, including hospital at home services^11^ and urgent community response teams.^12^ To optimize these approaches, it is important to understand which individuals are the highest users of emergency care.

To address this gap, a systematic review of international evidence was conducted. This review aimed to: (i) quantify emergency hospital attendances (defined as visits to the Emergency Department) and admissions (defined as subsequent admission to hospital) from the older population receiving different types of social care; and (ii) identify which social care setting made the greatest contribution of emergency hospital attendances and admissions.

## Methods

The protocol for this systematic review is registered on PROSPERO (ID CRD42022319784). The methods are reported in accordance with the PRISMA guidance and checklist.^13^

### Search strategy

A search strategy was developed and refined for the population (older people aged 60+), exposure (the type of social care received) and the two outcomes of interest (average number of emergency hospital attendances and admissions per person per year). Searches were restricted to observational studies published from 2012 onwards in high-income countries. Searches were carried out in MEDLINE, EMBASE (via OVID), CINAHL (via EBSCO), Health Management Information Consortium (HMIC), Scopus and SSCI Online (via Web of Science). Three sources of grey literature were also searched: The Health Foundation, The King’s Fund and Nuffield Trust. Searches were conducted in February 2022.

### Review criteria

Titles and abstracts of all search records were screened for relevance. The full texts of selected records were retrieved and assessed against the review criteria (Table 1). In both stages of screening, all records were screened independently by the primary researcher and one co-author; disagreements were resolved through consensus. Records were managed in Rayyan, an online software platform to manage screening in systematic reviews.^14^

**Table 1:** Systematic review criteria

**Parameter**	**Inclusion criteria**	**Exclusion criteria**
Population	Older people, aged 60+, who received a form of social care	Studies which exclude individuals who did not receive emergency care
Exposure (Type of social care received)	Type of social care received, including nursing homes, residential homes (without nursing), home-based social care (domiciliary care services referred to as ‘home care’), supported living services (referred to as ‘assisted living’) and support from an informal (not paid-for) carer, such as a relative or friend	Studies based in an Emergency Department setting reporting the proportion of individuals receiving various types of social care
Comparator	Any comparator (studies comparing multiple settings of care) and no comparator (studies in a single setting of care)	
Outcomes	Two outcomes are included: (i) the average number of emergency hospital attendances per person per year and (ii) the average number of emergency hospital admissions per person per year Studies which reported the number of emergency hospital attendances and the number of emergency hospital admissions, in addition to the total population size, were included as the desired outcomes could be derived	

### Data extraction

A data extraction form was developed, piloted and refined. Study characteristics (location, study period), population attributes (age, size of population, diagnoses, number of co-morbidities and proxy measures of care needs), exposures and outcomes of interest (including outcome definitions and 95% confidence intervals) were extracted where available, into a Microsoft Excel spreadsheet by one researcher. Data extraction was checked for accuracy by a second researcher for 50% of studies.

### Quality assessment

As this review is focused on observational studies, study quality was assessed using the National Institute of Health Quality Assessment’s Tool for Observational Cohort and Cross-Sectional Studies. One researcher assessed the quality of each study using 10 relevant domains of this tool, noting whether each statement was given ‘yes’, ‘no’, ‘cannot determine’ or ‘not applicable’ answers.

### Synthesis

A narrative synthesis was conducted, supported by forest plots. It was not possible to pool data for meta-analysis, due to heterogeneity in study design. The forest plots were used to illustrate the average number of emergency attendances and admissions per person per year, across different social care settings. Studies where Poisson confidence intervals could be derived were included in the plots. The average number of emergency attendances and admissions per person was derived for some studies (indicated in Table 2) by dividing the number of emergency hospital attendances or admissions by the study population size. In some instances, the number of emergency hospital attendances or admissions was derived using the reported percentage of the population who had an emergency attendance or admission. Data extracted where the study period was shorter or longer than 12 months was adjusted, to estimate the number of attendances or admissions over 12 months. Each forest plot was assessed visually to describe how emergency attendances or admissions varied between social care settings. Possible causes of heterogeneity between studies were explored by mapping differences in study design and study populations. Outliers were assessed on an individual basis, to identify any possible drivers of variation.

**Table 2:** Summary of all study characteristics

**Paper**	**Population characteristics**				**Study methodology**							**Indicators of level of need reported?**
	**County**	**Sample size—analysed**	**Average age**	**Health-related inclusion criteria**	**Study period**	**Study setting**	**Study design**	**Derived data**	**Is attendance & admission mutually exclusive?**	**Are repeated attendances or admissions included?**	**Are temporary residents included?**	
Amador 2014	England	133	86.2	Dementia	12 months	6 residential homes	Prospective cohort	Rate of attendance/admission	Yes	No	Yes	Co-morbidities
Bardsley 2012	England	133 055	82.2	None	12 months	Health and local authority funded social care in four areas	Retrospective cohort	Number of attendances/admissions	Yes	Yes	No	None
Blackburn 2016	USA	2582	80	None	21 months	Nursing homes, home health recipients	Matched retrospective cohort	Number of attendances/admissions	No	Yes	Yes	Mobility, toileting independence
de Souto Barreto 2013	France	5684	87	53.2% of sample had dementia/ suspected dementia	12 months	175 nursing homes	Cross-sectional	Rate of attendance/admission	No	No	No	Activities of Daily Living score, number of diseases
Dubucs 2018	France	5926	86	None	12 months	175 nursing homes	Cross-sectional	Rate of attendance/admission	No	Yes	No	None
Fassmer 2020	Germany	1665	80.5	None	24 months	Nursing homes	Retrospective cohort	No	Yes	Yes	No	Level of care dependency, co-morbidities
Givens 2012	USA	323	85.3	Advanced dementia	18 months	Nursing homes	Prospective cohort	Rate of attendance/admission	Yes	Yes	No	None
Gruneir 2016	Canada	71 780	84.4	None	12 months	Nursing homes	Retrospective cohort	Rate of attendance/admission	No	No	No	Activities of daily living score, cognitive performance scale
Hongli 2018	Singapore	202	78.9	None	12 months	1 nursing home	Retrospective cohort	No	Yes	Yes	No	Functional status
Hua 2021	USA	293 336	Not reported (sample are aged 65+)	None	12 months	Assisted living facilities	Retrospective cohort	Population size	No	Yes	Yes	None
Inacio 2021	Australia	116 192	85 (median age)	None	48 months	Residential aged care facilities	Retrospective cohort	N/A – not used in analysis	No	No	No	Dementia, frailty
Kihlgren 2014	Sweden	719	85.8	None	12 months	Nursing homes	Cross-sectional follow up	Rate of attendance/admission	No	Yes	Yes	Cognitive performance scale, number of diseases
Kirsebom 2014	Sweden	431	87	None	9 months	Nursing homes	Retrospective cohort	N/A – not used in analysis	Yes	Yes	Yes	None
LaMantia 2016	USA	4491	79.6	None	12 months	Nursing homes	Retrospective cohort	N/A – not used in analysis	Yes	No	No	Dementia severity
McGregor 2014	Canada	13 140	83.1	None	36 months	Nursing homes	Retrospective cohort	Rate of attendance/admission	No	Yes	Yes	None
McGregor 2014	Canada	13 051	Assisted living: 81.5 Nursing home: 83.1	None	36 months	Nursing homes, assisted living	Retrospective cohort	No	No	Yes	No	None
Mondor 2017	Canada	30 112	83	Dementia	12 months	Home care	Retrospective cohort	Rate of attendance/admission	Yes	No	No	Multimorbidities
Neufeld 2016	Canada	2061	Not reported	None	12 months	Home care	Cross sectional	N/A – not used in analysis	Yes	Yes	Yes	Activities of daily living hierarchy scale score
Stephens 2012	USA	132 753	Not reported	None	12 months	Nursing homes	Cross sectional	Rate of attendance/admission	No	No	Yes	Activities of daily living impairment, level of cognitive impairment
Stephens 2014	USA	112 412	Not reported	None	12 months	Nursing homes	Retrospective cohort	Number of attendances/admissions	Yes	Yes	Yes	Activities of daily living score
Walker 2014	England	63	81.3	Parkinson’s disease	24 months	Residential homes	Retrospective cohort	N/A – not used in analysis	Yes	Yes	No	Mobility
Wolters 2019	England	195 296	Residential home: 85.6 Nursing home: 84.7	None	12 months	Nursing homes, residential homes	Retrospective cohort	No	No	Yes	No	Charlson index, Elixhauser co-morbidity index, frailty-related conditions

## Results

**Fig. 1:**
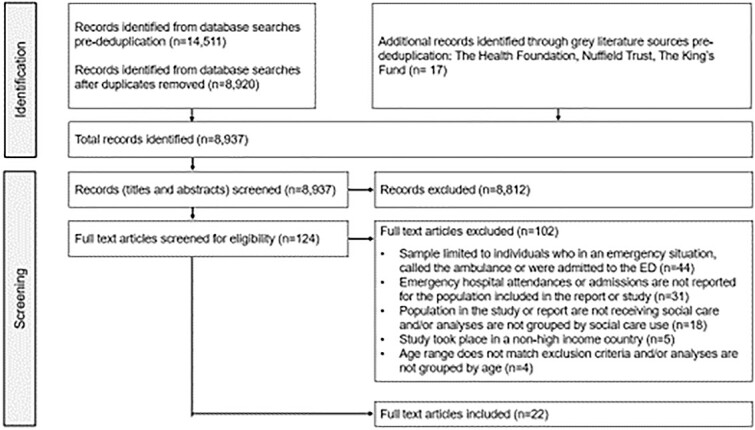
PRISMA flowchart, illustrating study selection process.

A total of 8937 records were identified for title and abstract screening, of which 124 were flagged for full-text screening. Twenty-two studies met the inclusion criteria for this review (Fig. 1).

### Quality assessment

Fifteen of the twenty-two included studies were rated as ‘good’ quality^15–29^ and seven studies were rated ‘fair’.^7^^,^^30–35^ Reasons for these ratings included omissions in reporting overall sample size (32) and confidence intervals for the metric of interest^7^^,^^30^^,^^32–35^; and studies with a participation rate below 50%.^31^ One study was excluded from the synthesis due to a short follow-up period.^35^ We judged that risk of reporting bias was low. The results of the quality assessment are included in the Supplementary Materials.

### Forest plots

Forest plots were generated to visualize the average number of hospital attendances and admissions, and associated 95% confidence intervals, for each study. Three high quality studies were excluded from the forest plots because the required outcome data were not reported.^24^^,^^28^^,^^29^ One paper was excluded from the forest plot presenting emergency hospital attendances due to missing data, however, it was possible to include this study in the forest plot pertaining to emergency hospital admissions.^23^

### Emergency hospital attendances

The average number of emergency hospital attendances per person per year was reported or derived in 16 studies. Of these 16 studies, 4 studies compared attendances between 2 or more social care settings.^7^^,^^15^^,^^26^^,^^31^ Each of these 4 studies compared emergency attendances in nursing homes with either residential homes,^7^ assisted living facilities^26^ or care received in the home.^15^^,^^31^ One of these studies did not distinguish between different types of care home.^15^ The remaining 12 studies reported attendances in a single setting of care: nursing homes^16–20^^,^^22^^,^^25^^,^^33^^,^^34^; residential homes^30^; home care^27^; and an assisted living facility.^21^ All 16 studies were judged to have a low risk of bias.

**Fig. 2:**
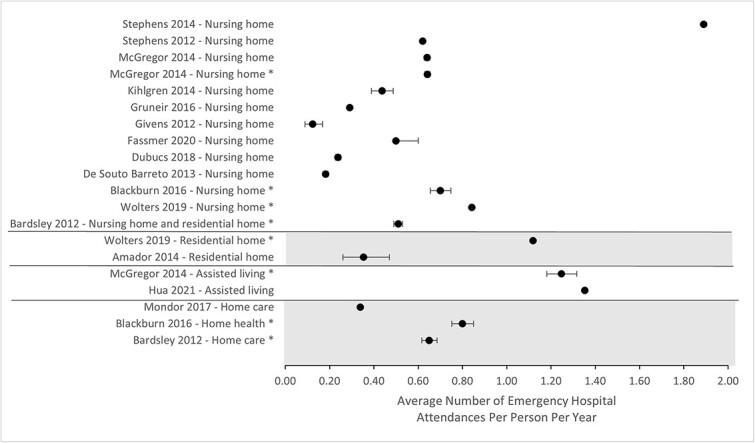
Forest plot illustrating the average number of emergency hospital attendances per person per year and calculated 95% confidence intervals for each included study. Studies marked with an asterisk (*) compared two or more settings of social care.

Rates of emergency hospital attendance were highest in assisted living facilities and lowest in nursing homes (Fig. 2). However, there was high variation in the average number of emergency hospital attendances, both between different settings of care and within the same setting of care. Reasons for this heterogeneity may be explained by differences in study populations and study design. Analysis of the group of comparator studies offered another approach to compare different settings of care. Emergency hospital attendance was lower in nursing homes in comparison with residential homes 7, assisted living facilities 26 and home health services 31. Similarly, emergency hospital attendances were lower from care homes compared with home care services 15. Reported differences in hospital attendance rates were small, with differences between care settings ranging from 0.1 to 0.61 additional hospital attendances per person per year.

### Emergency hospital admissions

**Fig. 3:**
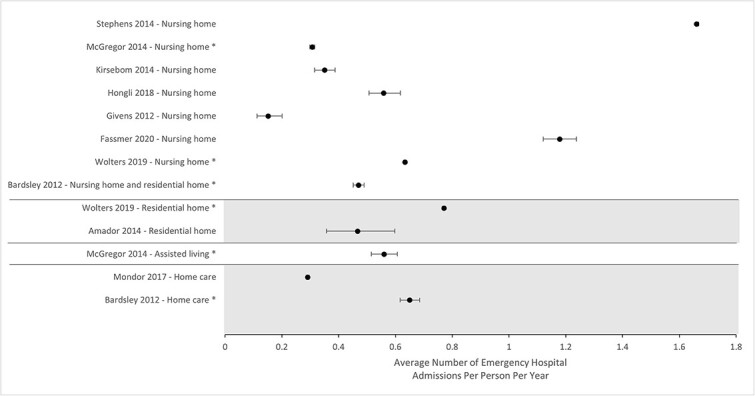
Forest plot illustrating the average number of emergency hospital admissions per person per year and calculated 95% confidence intervals for each included study. Studies marked with an asterisk (*) compared two or more settings of social care.

Nine studies reported the average number of emergency hospital admissions per person per year. Three studies compared admissions in nursing homes against residential homes,^7^ assisted living facilities^26^ or home care services.^15^ One study did not distinguish between different types of care home.^15^ Seven studies described emergency admissions in a single care setting: nursing homes,^18^^,^^19^^,^^23^^,^^32^^,^^34^ residential homes^30^ and home care.^27^ All studies were judged to have a low risk of bias. The average number of emergency hospital admissions per person per year for each study is presented in Figure 3.

We found no clear relationship between the number of emergency hospital admissions and settings of social care. Most of the variation seen in Figure 3 is driven by two studies (Stephens 2014 and Fassmer 2020), however closer inspection of these studies did not indicate what may be driving a higher number of emergency hospital admissions within these populations. Analysis of the group of comparator studies indicated that emergency admissions are lower from nursing homes compared with residential homes^7^ and assisted living facilities.^26^ Admissions were also found to be lower from care homes than in home care settings.^15^

### Heterogeneity between studies

The characteristics of each study are mapped out in Table 2. An assessment of the differences in study design and population characteristics was undertaken, to understand if there are factors other than setting of care which may be driving differences in the reported rates of emergency hospital attendance or admission.

Study populations varied based on their inclusion or exclusion of ‘short-term’ social care recipients. Some study populations were selected based on a diagnosis of dementia or cognitive impairment; others included all care recipients. Crucially, the included studies took place across eight countries, each with different systems of health and social care. Inconsistency in the reporting of population characteristics meant that, unfortunately, it was not possible to explore whether heterogeneity in the care needs (e.g. co-morbidities or pre-existing advanced care plans) of each study population may explain the variation in emergency admissions and attendances.

The measurement of the outcomes varied between studies. Some studies distinguished between emergency hospital attendances and admissions, while other studies did not. Higher emergency hospital attendance was expected in 10 studies that included individuals who were subsequently admitted to hospital in their estimates of emergency attendance. Furthermore, some studies did not account for *repeat* Emergency Department attendances and are therefore likely to underestimate emergency hospital attendance.

The lack of comparability between studies limits the conclusions we can draw. However, illustrating the study data in forest plots can help to reveal potential trends in emergency hospital attendance or admission across each setting of care.

## Discussion

### Summary of findings

This review explores existing data on the emergency care use of older people living in different settings of social care. We found limited data in care settings outside of nursing homes, including individuals receiving care at home or in assisted living facilities. No data were found pertaining to informal care. Although data are limited, there appears to be a higher number of emergency hospital attendances in assisted living facilities compared with nursing homes, suggesting that emergency attendance may vary according to the level of nursing input provided within the care setting. However, variation in emergency attendance was high between studies taking place within the same setting. Less variation was observed between emergency admissions across different settings of care, suggesting that once a threshold of severity is reached, there is consistency in hospital decision-making to admit patients.

### Comparison with other work

Previous research has suggested that the presence of registered nurses within a social care setting can moderate ambulance call rates and hospital admissions.^25^^,^^36^^,^^37^ Recent research demonstrated that people with dementia in the last year of their life, who live in a local authority with more nursing home beds, have fewer visits to the Emergency Department than people living in local authorities with a lower number of nursing home beds.^38^ Our review suggests that emergency hospital attendances are lower in nursing homes in comparison with other care settings, including residential homes,^7^ assisted living facilities^26^ and home care services.^15^^,^^31^ This finding may be surprising, as one would expect individuals residing in an assisted living facility to have fewer complex care needs than individuals living in a nursing home, for example, and therefore one may be expect residents of assisted living facilities to require less hospital care. Previous authors have suggested several reasons why Emergency Department attendance is lower amongst nursing home residents in comparison with other care settings. Older people with higher health instability and cognitive impairment, who are more likely to live in a nursing home, are more likely to have a ‘do not hospitalize’ order, which consequently leads to lower rates of hospitalization.^39^ Another explanation is that health needs may be detected earlier in care settings with nursing. Similarly, staff in care facilities with access to nursing may be better equipped to manage patient health within the facility, and therefore may be less reliant on emergency care.^7^

### Implications

Our review highlights a stark lack of data on emergency care use in social care settings outside of care homes. Of the six included studies that reported on emergency care use in settings other than care homes, five studies took place in the USA or Canada, where use of a minimum dataset in social care is common. One study highlights the potential of conducting analyses of linked health and social care data in England. However this study is limited to local authority-funded care and does not account for those who self-fund their care.^15^ Uptake of a minimum dataset in England, linked to health data, would enable further exploration on the needs and outcomes of these populations. Our review also highlights inconsistencies in reporting of population characteristics between studies. Consistent reporting of care needs would facilitate comparisons between studies.

Evidence presented in our review suggests that older people living in nursing homes visit the Emergency Department marginally less often than older people in other social care settings. However, an older person’s place of residence does not appear to influence the likelihood of them being admitted to hospital, after visiting the Emergency Department. Our review found that individuals who visit the Emergency Department but are not subsequently admitted to hospital are more likely to be living in social care settings without registered nurses on site, such as assisted living facilities. These individuals may benefit the most from targeting of community-based resources, such as district nurse and primary care input.

### Strengths and limitations

A strength of this review is its inclusion of all social care settings, enabling us to identify a gap in our understanding of emergency care use in assisted living facilities and home care services. It is important that more research is conducted in these care settings, as we are becoming increasingly reliant on these settings of care to meet the needs of our older population. We also assessed outcomes specific to emergency care, which are neglected in the current literature. Drawing conclusions from our analysis was challenging, due to variation in study population characteristics and outcome definitions. Although variation in levels of emergency care use within a setting of social care is to be expected,^20^^,^^23^^,^^25^ some variation may be driven by differences in study design. A limitation of our approach was that differences in study design are not accounted for in our analysis. Unfortunately, this was not possible due to multiple drivers of variation and a relatively small group of included studies. Future evidence on emergency care use would benefit from consistency in outcome definitions and study design.

Unfortunately, it was not possible to account for differences in the care needs of each study population in our review. We extracted data for a variety of proxy measures of care need, but found that these metrics were not consistently reported. One of our included studies demonstrated that a group of individuals with mild cognitive impairment had greater use of emergency care than a group with severe cognitive impairment.^34^ This finding may reflect the findings of our review, as older people with severe cognitive impairment have higher social care needs and are therefore more likely to require nursing care than individuals with mild cognitive impairment. Further research would be needed to clarify how an individual’s level of care need impacts their use of emergency care. It is important to note that the included studies were published between 2012 and 2020, with data collected prior to the COVID-19 pandemic. It would be interesting to compare the results of this review with future studies, to determine how changes to the healthcare system such as increased hospital at home^40^ have impacted emergency hospital attendances.

## Conclusions

This review highlights data gaps in the emergency care use of older people in social care, particularly in settings other than care homes. Despite data being limited, this review suggests that the level of nursing input within a social care setting moderates its contribution of emergency hospital attendances, whereby older people with access to nursing care are less likely to visit the Emergency Department than older people residing in assisted living facilities.

## Data availability

The authors confirm that the data supporting the findings of this study are available within the article and its supplementary materials.

## Supplementary Material

BMB_Submission_Supplementary_Data_Revised_ldad033
