# Supplementary material for: Variations in older people’s emergency care use by social care setting: a systematic review of international evidence
Source: Br Med Bull. 2023 Dec 18;149(1):32–44. doi: 10.1093/bmb/ldad033 (PMC10938536; doi:10.1093/bmb/ldad033)
Supplement: BMB_Submission_Supplementary_Data_Revised_ldad033 [file bmb_submission_supplementary_data_revised_ldad033.docx]

# **Supplementary data**

## Search Strategy

**MEDLINE**

Ovid MEDLINE(R) <1996 to January Week 4 2022>

1        elder*.mp.

2        senior*.mp.

3        veteran*.mp.

4        retire*.mp.

5        exp Aged/

6        1 or 2 or 3 or 4 or 5

7        social care.mp.

8        long-term care.mp.

9        green house.mp.

10        extra care housing.mp.

11        sheltered accommodation.mp.

12        care service*.mp.

13        nursing home*.mp.

14        care home*.mp.

15        veteran* home*.mp.

16        retire* home*.mp.

17        convalescent home*.mp.

18        home care.mp.

19        residential care.mp.

20        domiciliary care.mp.

21        elder* care.mp.

22        respite care.mp.

23        community care.mp.

24        home nursing.mp.

25        home help.mp.

26        assisted living.mp.

27        supported living.mp.

28        retire* facilit*.mp.

29        nursing facilit*.mp.

30        care facilt*.mp.

31        retirement communit*.mp.

32        retirement housing.mp.

33        retirement village.mp.

34        carer*.mp.

35        caregiver*.mp.

36        Long-Term Care/

37        Home Care Services, Hospital-Based/

38        exp Home Nursing/

39        Home Health Nursing/

40        Senior Centers/

41        Group Homes/

42        Assisted Living Facilities/

43        Homes for the Aged/

44        Homemaker Services/

45        exp Nursing Homes/

46        Home Care Agencies/

47        Housing for the Elderly/

48        Health Services for the Aged/

49        Home Health Aides/

50        7 or 8 or 9 or 10 or 11 or 12 or 13 or 14 or 15 or 16 or 17 or 18 or 19 or 20 or 21 or 22 or 23 or 24 or 25 or 26 or 27 or 28 or 29 or 30 or 31 or 32 or 33 or 34 or 35 or 36 or 37 or 38 or 39 or 40 or 41 or 42 or 43 or 44 or 45 or 46 or 47 or 48 or 49

51        emergency hospital.mp.

52        emergency service*.mp.

53        emergency care.mp.

54        emergency unit*.mp.

55        emergency room.mp.

56        emergency department*.mp.

57        emergency ward*.mp.

58        "accident and emergency".mp.

59        ambulance.mp.

60        exp Emergency Service, Hospital/

61        Patient Admission/

62        51 or 52 or 53 or 54 or 55 or 56 or 57 or 58 or 59 or 60 or 61

63        6 and 50 and 62

64        limit 63 to (english language and yr="2012 -Current")

**EMBASE**

Ovid MEDLINE(R) <1996 to January Week 4 2022>

1        elder*.mp.

2        senior*.mp.

3        veteran*.mp.

4        retire*.mp.

5        exp Aged/

6        1 or 2 or 3 or 4 or 5

7        social care.mp.

8        long-term care.mp.

9        green house.mp.

10        extra care housing.mp.

11        sheltered accommodation.mp.

12        care service*.mp.

13        nursing home*.mp.

14        care home*.mp.

15        veteran* home*.mp.

16        retire* home*.mp.

17        convalescent home*.mp.

18        home care.mp.

19        residential care.mp.

20        domiciliary care.mp.

21        elder* care.mp.

22        respite care.mp.

23        community care.mp.

24        home nursing.mp.

25        home help.mp.

26        assisted living.mp.

27        supported living.mp.

28        retire* facilit*.mp.

29        nursing facilit*.mp.

30        care facilt*.mp.

31        retirement communit*.mp.

32        retirement housing.mp.

33        retirement village.mp.

34        carer*.mp.

35        caregiver*.mp.

36        Long-Term Care/

37        Home Care Services, Hospital-Based/

38        exp Home Nursing/

39        Home Health Nursing/

40        Senior Centers/

41        Group Homes/

42        Assisted Living Facilities/

43        Homes for the Aged/

44        Homemaker Services/

45        exp Nursing Homes/

46        Home Care Agencies/

47        Housing for the Elderly/

48        Health Services for the Aged/

49        Home Health Aides/

50        7 or 8 or 9 or 10 or 11 or 12 or 13 or 14 or 15 or 16 or 17 or 18 or 19 or 20 or 21 or 22 or 23 or 24 or 25 or 26 or 27 or 28 or 29 or 30 or 31 or 32 or 33 or 34 or 35 or 36 or 37 or 38 or 39 or 40 or 41 or 42 or 43 or 44 or 45 or 46 or 47 or 48 or 49

51        emergency hospital.mp.

52        emergency service*.mp.

53        emergency care.mp.

54        emergency unit*.mp.

55        emergency room.mp.

56        emergency department*.mp.

57        emergency ward*.mp.

58        "accident and emergency".mp.

59        ambulance.mp.

60        exp Emergency Service, Hospital/

61        Patient Admission/

62        51 or 52 or 53 or 54 or 55 or 56 or 57 or 58 or 59 or 60 or 61

63        6 and 50 and 62

64        limit 63 to (english language and yr="2012 -Current")

**CINAHL**

HMIC Health Management Information Consortium <1979 to November 2021>

1        exp older people/ or exp geriatrics/ or exp gerontology/ or exp old age/

2        elder*.mp.

3        senior*.mp.

4        veteran*.mp.

5        retire*.mp.

6        1 or 2 or 3 or 4 or 5

7        social care.mp.

8        long-term care.mp.

9        green house.mp.

10        extra care housing.mp.

11        sheltered accommodation.mp.

12        care service*.mp.

13        nursing home*.mp.

14        care home*.mp.

15        veteran* home*.mp.

16        retire* home*.mp.

17        convalescent home*.mp.

18        home care.mp.

19        residential care.mp.

20        domiciliary care.mp.

21        elder* care.mp.

22        respite care.mp.

23        community care.mp.

24        home nursing.mp.

25        home help.mp.

26        assisted living.mp.

27        supported living.mp.

28        nursing facilit*.mp.

29        care facilit*.mp.

30        retirement communit*.mp.

31        retirement housing.mp.

32        retirement village.mp.

33        carer*.mp.

34        caregiver*.mp.

35        exp nursing homes/ or exp old peoples homes/ or exp residential care/ or exp retirement communities/

36        exp Home care/

37        exp home nursing/

38        exp extra care housing/ or exp health services for elderly people/ or exp health services for older people/ or exp "residential care of the elderly"/ or exp "residential care of the older"/ or exp sheltered housing/

39        exp Group homes/

40        7 or 8 or 9 or 10 or 11 or 12 or 13 or 14 or 15 or 16 or 17 or 18 or 19 or 20 or 21 or 22 or 23 or 24 or 25 or 26 or 27 or 28 or 29 or 30 or 31 or 32 or 33 or 34 or 35 or 36 or 37 or 38 or 39

41        (emergency hospital or emergency room or emergency service* or emergency care or emergency unit* or emergency department* or emergency ward* or "accident and emergency" or ambulance).mp.

42        exp emergency health services/

43        exp patient admission/

44        41 or 42 or 43

45        6 and 40 and 44

46        limit 45 to yr="2012 -Current"

**Scopus and SSCI**

Searches conducting using the following key words:

(“elder*” OR “senior*” OR “veteran*” OR “retire*”) AND (“Social care” OR “long-term care” OR “Green House” OR “Extra care housing” OR “Sheltered accommodation” OR “Care service*” OR “Nursing home*” OR “Care home*” OR “Veteran* home*” OR “Retire* home*” OR “Convalescent home*” OR “Home care” OR “Residential care” OR “Domiciliary care” OR “Elder* care” OR “Respite care” OR “Community care” OR “Home nursing” OR “Home help” OR “Assisted living” OR “Supported living” OR “Retire* facilit*” OR “Nursing facilit*” OR “Care facilit*” OR “Retirement communit*” OR “Retirement housing” OR “Retirement village” OR “carer*” OR “caregiver*”) AND ("Emergency hospital” OR ”Emergency service* OR “Emergency care” OR “Emergency unit* OR “Emergency room” OR “Emergency Department* OR “Emergency Ward*” OR “Accident and emergency” OR “Ambulance”)

## Quality assessment

Each study was assessed using elements of the NIHR Quality Assessment Tool for Observational Cohort and Cross-Sectional Studies, as outlined below. There are a set of answers to chose for each question: yes, no, CD (cannot determine), NR (not reported), NA (not applicable). Some elements of the assessment tool were not applicable to this study and therefore were not included in our quality assessment.

| **Paper** | **NIHR Quality Assessment Tool for Observational Cohort and Cross-Sectional Studies** | | | | | | | | | | **Additional considerations related to study quality** | **Rating (Good, Fair or Poor)** |
| --- | --- | --- | --- | --- | --- | --- | --- | --- | --- | --- | --- | --- |
|  | **1: Was the research question or objective in this paper clearly stated?** | **2: Was the study population clearly specified and defined?** | **3: Was the participation rate of eligible persons at least 50%?** | **4: Were all the subjects selected or recruited from the same or similar populations (including the same time period)? Were inclusion and exclusion criteria for being in the study prespecified and applied uniformly to all participants?** | **5: Was a sample size justification, power description, or variance and effect estimates provided?** | **7: Was the timeframe sufficient so that one could reasonably expect to see an association between exposure and outcome if it existed?** | **9: Were the exposure measures (independent variables) clearly defined, valid, reliable, and implemented consistently across all study participants?** | **11: Were the outcome measures (dependent variables) clearly defined, valid, reliable, and implemented consistently across all study participants?** | **13: Was loss to follow-up after baseline 20% or less?** | **14: Were key potential confounding variables measured and adjusted statistically for their impact on the relationship between exposure(s) and outcome(s)?** |  |  |
| Amador 2014 | Yes | Yes | Yes | Yes | No | Yes | Yes | Yes | NA | NA |  | Fair |
| Bardsley 2012 | Yes | Yes | Yes | Yes | Yes | Yes | Yes | Yes | NA | Yes |  | Good |
| Blackburn 2016 | Yes | Yes | NA | Yes | Yes | Yes | No | Yes | Yes | Yes | At the end of follow up period, many individuals were no longer receiving the stated form of social care | Fair |
| de Souto Barreto 2013 | Yes | Yes | Yes | Yes | No | Yes | Yes | Yes | Yes | Yes |  | Good |
| Dubucs 2018 | Yes | Yes | Yes | Yes | Yes | Yes | Yes | Yes | NA | Yes |  | Good |
| Fassmer 2020 | Yes | Yes | Yes | Yes | Yes | Yes | Yes | Yes | Yes | No |  | Good |
| Givens 2012 | Yes | Yes | Yes | Yes | NA | Yes | Yes | Yes | Yes | Yes |  | Good |
| Gruneir 2016 | Yes | Yes | Yes | Yes | Yes | Yes | Yes | Yes | Yes | Yes |  | Good |
| Hongli 2018 | Yes | No | CD | Yes | No | Yes | Yes | Yes | CD | No |  | Fair |
| Hua 2021 | Yes | Yes | Yes | Yes | Yes | Yes | Yes | Yes | Yes | Yes |  | Good |
| Inacio 2021 | Yes | Yes | Yes | Yes | No | No | Yes | Yes | Yes | NA |  | Fair |
| Kihlgren 2014 | Yes | Yes | Yes | Yes | No | Yes | Yes | Yes | Yes | NA |  | Good |
| Kirsebom 2014 | Yes | Yes | Yes | Yes | Yes | Yes | Yes | Yes | Yes | NA |  | Good |
| LaMantia 2016 | Yes | Yes | Yes | No | Yes | Yes | Yes | Yes | Yes | NA |  | Good |
| McGregor 2014  (Nursing home only) | Yes | Yes | Yes | Yes | Yes | Yes | Yes | Yes | Yes | Yes |  | Good |
| McGregor 2014  (Nursing homes &  assisted living) | Yes | Yes | Yes | Yes | Yes | Yes | Yes | Yes | Yes | NA |  | Good |
| Mondor 2017 | Yes | Yes | Yes | Yes | No | Yes | Yes | Yes | Yes | No |  | Good |
| Neufeld 2016 | Yes | Yes | Yes | Yes | Yes | Yes | Yes | Yes | Yes | NA |  | Good |
| Stephens 2012 | Yes | Yes | Yes | Yes | No | Yes | Yes | Yes | Yes | NA |  | Fair |
| Stephens 2014 | Yes | Yes | Yes | Yes | No | Yes | Yes | Yes | Yes | NA |  | Fair |
| Walker 2014 | Yes | Yes | Yes | Yes | Yes | Yes | Yes | Yes | Yes | NA |  | Good |
| Wolters 2019 | Yes | Yes | CD | Yes | No | Yes | Yes | Yes | NA | No | The estimated population size was lower than the 2011 census, so a corrective factor was applied across calculations | Fair |

## Statistical syntheses: emergency hospital attendances

| **Author and setting of care** | **Average number of A&E visits per person per year** | **Lower Confidence Interval** | **Upper Confidence Interval** |
| --- | --- | --- | --- |
| Bardsley 2012 - Home care * | 0.65 | 0.62 | 0.69 |
| Mondor 2017 - Home care | 0.34 | 0.33 | 0.35 |
| Blackburn 2016 - Home health * | 0.80 | 0.75 | 0.85 |
| Hua 2021 - Assisted living | 1.35 | 1.35 | 1.36 |
| McGregor 2014 - Assisted living * | 1.25 | 1.18 | 1.32 |
| Wolters 2019 - Residential home * | 1.12 | 1.11 | 1.12 |
| Amador 2014 - Residential home | 0.35 | 0.26 | 0.47 |
| Bardsley 2012 - Nursing home and residential home * | 0.51 | 0.49 | 0.53 |
| Wolters 2019 - Nursing home * | 0.84 | 0.84 | 0.85 |
| Blackburn 2016 - Nursing home * | 0.70 | 0.66 | 0.75 |
| McGregor 2014 - Nursing home * | 0.64 | 0.63 | 0.65 |
| De Souto Barreto 2013 - Nursing home | 0.18 | 0.17 | 0.19 |
| Dubucs 2018 - Nursing home | 0.24 | 0.23 | 0.25 |
| Fassmer 2020 - Nursing home | 0.50 | 0.50 | 0.60 |
| Givens 2012 - Nursing home | 0.12 | 0.09 | 0.17 |
| Gruneir 2016 - Nursing home | 0.29 | 0.29 | 0.29 |
| Kihlgren 2014 - Nursing home | 0.44 | 0.39 | 0.49 |
| McGregor 2014 - Nursing home | 0.64 | 0.63 | 0.65 |
| Stephens 2012 - Nursing home | 0.62 | 0.62 | 0.62 |
| Stephens 2014 - Nursing home | 1.89 | 1.88 | 1.90 |

*Studies marked with an asterisk (*) include more than one setting of care. Each record is grouped by setting of care and arranged alphabetically.*

## Statistical syntheses: emergency hospital admissions

| **Author and setting of care** | **Average number of Emergency Admissions per person per year** | **Lower Confidence Interval** | **Upper Confidence Interval** |
| --- | --- | --- | --- |
| Bardsley 2012 - Home care * | 0.65 | 0.62 | 0.69 |
| Mondor 2017 - Home care | 0.29 | 0.28 | 0.30 |
| McGregor 2014 - Assisted living * | 0.56 | 0.52 | 0.61 |
| Wolters 2019 - Residential home * | 0.77 | 0.77 | 0.78 |
| Amador 2014 - Residential home | 0.47 | 0.36 | 0.60 |
| Bardsley 2012 - Nursing home and residential home * | 0.47 | 0.45 | 0.49 |
| McGregor 2014 - Nursing home * | 0.31 | 0.30 | 0.32 |
| Wolters 2019 - Nursing home * | 0.63 | 0.63 | 0.64 |
| Fassmer 2020 - Nursing home | 1.18 | 1.12 | 1.24 |
| Givens 2012 - Nursing home | 0.15 | 0.11 | 0.20 |
| Hongli 2018 - Nursing home | 0.56 | 0.51 | 0.62 |
| Kirsebom 2014 - Nursing home | 0.35 | 0.32 | 0.39 |
| Stephens 2014 - Nursing home | 1.66 | 1.66 | 1.67 |

*Studies marked with an asterisk (*) include more than one setting of care. Each record is grouped by setting of care and arranged alphabetically.*
